# Different Performances of Different Intelligent Algorithms for Solving FJSP: A Perspective of Structure

**DOI:** 10.1155/2018/4617816

**Published:** 2018-09-02

**Authors:** Xiao-qiu Shi, Wei Long, Yan-yan Li, Yong-lai Wei, Ding-shan Deng

**Affiliations:** School of Manufacturing Science and Engineering, Sichuan University, Chengdu 610000, China

## Abstract

There are several intelligent algorithms that are continually being improved for better performance when solving the flexible job-shop scheduling problem (FJSP); hence, there are many improvement strategies in the literature. To know how to properly choose an improvement strategy, how different improvement strategies affect different algorithms and how different algorithms respond to the same strategy are critical questions that have not yet been addressed. To address them, improvement strategies are first classified into five basic improvement strategies (five structures) used to improve invasive weed optimization (IWO) and genetic algorithm (GA) and then seven algorithms (S1–S7) used to solve five FJSP instances are proposed. For the purpose of comparing these algorithms fairly, we consider the total individual number (TIN) of an algorithm and propose several evaluation indexes based on TIN. In the process of decoding, a novel decoding algorithm is also proposed. The simulation results show that different structures significantly affect the performances of different algorithms and different algorithms respond to the same structure differently. The results of this paper may shed light on how to properly choose an improvement strategy to improve an algorithm for solving the FJSP.

## 1. Introduction

Brucker and Schlie proposed the flexible job-shop scheduling problem (FJSP) [1] for the first time in 1990, in which every operation can be processed on more than one machine. Therefore, FJSP is more difficult than the classical job-shop scheduling problem (JSP), which is a NP-hard problem [2] in which every operation can be processed on just one machine. Owing to the complexity of FJSP, many researchers have used different intelligent algorithms to solve it in recent years. Most intelligent algorithms are first proposed to solve the continuous optimization problem; however, FJSP is a classical combinatorial optimization problem. Therefore, these algorithms must be improved before solving it. For example, Lu et al. [3] proposed a multiobjective discrete virus optimization algorithm (MODVOA), an improved virus optimization algorithm, to solve FJSP, demonstrating that the proposed MODVOA can achieve better performance than other algorithms. Using specially designed discrete operators to produce new individuals, Huang and Tian [4] presented a modified discrete particle swarm optimization to solve FJSP. Gao et al. [5] proposed an effective discrete harmony search (DHS) algorithm for this purpose. Moreover, several local search methods were embedded to enhance DHS's local exploitation capability. Computational results and comparisons demonstrated the efficiency of the proposed DHS. Li et al. [6] used a discrete strategy to improve the artificial bee colony (DABC) algorithm, and a novel DABC algorithm was proposed to solve the multiobjective FJSP. Zhang and Wen [7] proposed a multipopulation genetic algorithm (GA) for the multiobjective FJSP, and it exhibits far better performance than other algorithms. Xing et al. [8] presented a multipopulation interactive coevolutionary algorithm for solving FJSP. Its performance was evaluated using numerous benchmark instances. Chang and Liu [9] proposed a hybrid GA for solving the distributed and flexible job-shop scheduling problem and used the Taguchi method to optimize the GA parameters. Liu et al. [10] proposed a hybrid fruit fly optimization algorithm for solving FJSP and proved its performance with a case study. Wu and Wu [11] proposed a hybrid ant colony algorithm based on the 3D disjunctive graph model by combining the elitist ant system, max-min ant system, and the staged parameter control mechanism for solving FJSP. Using the GA and variable neighborhood search (VNS), Azzouz et al. [12] proposed a hybrid algorithm to solve FJSP, and the performance of the proposed algorithm was demonstrated by comparing its results with other methods. Zandieh et al. [13] proposed an improved imperialist competitive algorithm that was enhanced by simulated annealing to solve FJSP. Li and Gao [14] proposed an effective hybrid algorithm that hybridized the GA and tabu search (TS) for FJSP. Li et al. [15] proposed an effective hybrid TS algorithm (HTSA) for FJSP. A speedup local search method and a VNS were integrated into the HTSA, and they used some well-known benchmark instances to test it. Maroosi et al. [16] proposed a parallel-membrane-inspired harmony search for the purpose of increasing the diversity of the harmony search and improving the performance of the harmony search to solve FJSP, and their experimental results demonstrated the effectiveness of the proposed parallel algorithm.

As discussed above, we note that different authors have presented different improvement strategies, some of which are very complicated strategies that involve several algorithms or operators. They are usually enthusiastic about using more complicated improvement strategies to devise better algorithms; therefore, there are many improvement strategies, and the algorithms are becoming increasingly complicated. The complicated algorithms that exhibit better performance have been more or less obtained by trial and error. Thus, how different improvement strategies affect the performances of different algorithms and how different algorithms respond to the same improvement strategy are two critical questions that have not yet been reported in the literature. By addressing the two questions, we can properly choose an improvement strategy to improve an algorithm for solving FJSP.

To answer these two questions, we first classify the hundreds of improvement strategies available in the literature into five basic classifications corresponding to five basic improvement strategies, through which more complicated improvement strategies will be obtained. In an intelligent algorithm, many individuals included in a population evolve simultaneously. Essentially, improvement strategies decide the relationships among different algorithms or the relationships among different operators of different algorithms, so they also decide the relationships among individuals of an algorithm. Thus, an algorithm can be looked at as a complex system approximately consisting of connected individuals. An individual of a certain algorithm obtained through a certain improvement strategy has a particular way of communicating with other individuals, which means that the connections between individuals of different algorithms obtained through different improvement strategies are different. Thus, we naturally call the five basic improvement strategies five basic structures: discrete, multipopulation, mixed, parallel, and multistage structures. Discrete structure means that some discretization methods are used to improve an algorithm and the improvement strategies used in References [3–6] belong to this structure. Multipopulation structure means that more than one population is used to design an algorithm, and the improvement strategies used in References [7, 8] belong to this structure. This strategy is used to improve population diversity and avoid premature convergence. Mixed structure means that operators of an algorithm or its main idea are used in another algorithm; the improvement strategies used in References [9–15] belong to this structure. This structure may be the most frequently used improvement strategy in the literature. Parallel structure means that there are two or more different populations corresponding to two or more different algorithms in a newly obtained algorithm. Parallel structure, as in Reference [16], differs from multipopulation structure in that there is only one algorithm in multipopulation structure. A multistage structure is like the parallel structure in that they both use two or more different algorithms to obtain a new algorithm. However, they are different in that the two or more populations of a parallel structure are evolved simultaneously compared to the two or more populations of multistage structure evolving one after another. To the best of our knowledge, few papers on multistage structures as defined here exist in the literature. Thus, we use this multistage structure to obtain a novel multistage algorithm that will be described later.

We use the five basic structures to improve the GA and IWO, after which we obtain seven algorithms. As we all know, the GA is a well-known, widely used algorithm, and many researchers have used it to solve FJSP [17–19]. Conversely, there are fewer researchers who have used IWO to solve JSP, let alone FJSP. For example, Chen et al. [20], Zhou et al. [21], and Mishra et al. [22] used IWO to solve the permutation flow-shop scheduling problem, no-idle flow-shop scheduling problem, and JSP, respectively. Thus, we try to improve IWO and use it to solve FJSP in this paper.

We use the proposed seven algorithms to solve the five FJSP instances proposed in Reference [23], and the performance of these algorithms is illustrated to answer the two questions mentioned above. To compare these seven algorithms fairly, we consider the total individual number (TIN) in this paper. Traditionally, researchers [13, 24–26] frequently use efficiency and/or optimal value to evaluate different algorithms. However, there are some limitations without considering the different parameters of different algorithms. Regarding the efficiency, which means the total running time (or CPU time) of an algorithm, the computer language, the style of developing programs, the environment, and the parameters of an algorithm will influence the efficiency significantly. Regarding the optimal value, which means the best solution obtained by an algorithm, different algorithms that have different parameters find the same optimal value by searching different TINs, which are defined as the number of individuals used in an algorithm. For the standard GA, if every population has 100 individuals and the number of iterations is 100, then the TIN is 10,000, approximately. For IWO, if the number of iterations is also 100, the minimal population size is 10, the maximal population size is 100, the minimal seed size is 1, the maximal seed size is 5, and the TIN is 30,000. From this perspective, it is not fair if we just use optimal value and/or efficiency to evaluate the different intelligent algorithms. Therefore, we consider TIN, and several evaluation indexes based on TIN are presented in this paper. Different algorithms have different TINs obviously, because of different parameters. An intelligent algorithm is essentially a random search algorithm with some control strategies. Thus, the intelligent algorithm that has the larger TIN should have the better solution. In other words, the performance of an intelligent algorithm that obtains a better solution through a smaller TIN is better than other algorithms that obtain worse or equal solutions through a larger TIN.

In the process of decoding, a novel decoding algorithm that can obtain an active schedule is also proposed. Using computer simulations, the results show that different structures significantly affect different algorithms, and those different algorithms indeed have different responses to the same structure.

## 2. FJSP and Its Mathematical Model

FJSP has been formulated many times in the literature [15, 27]. The frequently used objectives are minimizing maximum completion time, minimizing maximum machine workload, and so on. We choose minimizing maximum completion time in this paper. The proposed mathematical model here is comparable to the model in [27], and the following assumptions are made:

1. The number of jobs and machines are known and fixed
2. The processing time of every operation is known and fixed
3. The processing order of operations for the same job is known and fixed
4. Every machine can be used at the beginning time and machine breakdowns are negligible
5. Materials to be used are prepared at the beginning time and loading times are negligible
6. The same operation can only be processed on the same machine at the same time and cannot be disrupted
7. Every machine can process at most one operation at the same time
8. The order of candidate operations of different jobs on the same machine is random

The mathematical model is as follows:

(1) $$\begin{array}{c} \min\;F_{\max}=\min_{i}\left(\max_{j}\left(F_{ij}\right)\right), \end{array}$$

(2) $$\begin{array}{c} \text{s}.\text{t}.\;J={\left\{J_{i}\right\}}_{i=1}^{n}, \end{array}$$

(3) $$\begin{array}{c} J_{i}={\left\{O_{ij}\right\}}_{j=1}^{n_{i}}, \end{array}$$

(4) $$\begin{array}{c} M={\left\{M_{k}\right\}}_{k=1}^{m}, \end{array}$$

(5) $$\begin{array}{c} F_{ij}-F_{i\left(j-1\right)}-P_{ijk}\times X_{ijk}\geq 0,\;\forall i,j,k, \end{array}$$

(6) $$\begin{array}{c} \sum_{k\in S_{ij}}X_{ijk}=1\;\wedge \;F_{ijk}-B_{ijk}=P_{ijk},\;\forall i,j, \end{array}$$

(7) $$\begin{array}{c} F_{i^{\prime }j^{\prime }k}\leq B_{ijk}\vee F_{ijk}\leq B_{i^{\prime }j^{\prime }k},\;\forall i^{\prime },j^{\prime }\neq i,j, \end{array}$$

(8) $$\begin{array}{c} X_{ijk}\in \left\{0,1\right\}. \end{array}$$

In this model, there is a set of *n* jobs that are processed on a set of *m* machines in the shop. *F*_*ij*_ and *F*_max_ in Equation (1) (that denotes the objective function) denote the finish time of *O*_*ij*_ (the *j*th operation of the *i*th job) and the maximal finish time of all jobs, respectively. In Equation (2), *J* denotes the job set and *J*_*i*_ the *i*th job, respectively, and *J* includes *n* jobs. In Equation (3), the number of operations of *J*_*i*_ is *n*_*i*_. In Equation (4), *M* denotes the machine set and *M*_*k*_ the *k*th machine, and *M* includes *m* machines. Inequity (5) ensures the correct processing order of operations for the same job, and *X*_*ijk*_ equals 1 when *O*_*ij*_ is processed on *M*_*k*_ and equals 0 otherwise. *P*_*ijk*_ denotes the processing time of *O*_*ij*_ on *M*_*k*_. *F*_*ijk*_ and *B*_*ijk*_ in Equation (6) (which ensures each operation can only be processed on one machine at the same time) denote the finish and start time of *O*_*ij*_ on *M*_*k*_, respectively, and the symbol “∧” denotes logical AND. *S*_*ij*_ denotes the machines on which *O*_*ij*_ can be processed. Inequity (7) ensures that every machine can process only one operation at a time and the symbol “∨” denotes logical OR. There is a FJSP instance which included three jobs and six machines shown in Table 1, where the number 0 denotes an operation that cannot be processed on a machine.

## 3. Proposed Seven Algorithms

After using the five basic structures to improve the GA and IWO, we obtain seven algorithms called S1–S7. For the purpose of comparing these algorithms fairly, we consider the TIN of an algorithm and several evaluation indexes based on TIN are presented. The first question is calculating the TIN of an algorithm according to its parameters, after which we can calculate other parameters of an algorithm (e.g., the number of iterations) when TINs are given. The steps of the seven algorithms and how to calculate their TINs are described in the following sections.

### 3.1. Discrete GA (S1)

S1 is obtained using a discrete structure to improve the GA. The discrete structure here exactly means integer encoding that will be described later. For the convenience of description, the steps of S1 are given as follows [28]:

*Step 1-1: Initialization*. Using integer encoding, some individuals are initialized randomly. These individuals are included in a population whose size (*P*_ga_) has been given in advance.

*Step 1-2: Decoding*. Using a novel decoding algorithm that will be described later, the fitness of each individual is obtained (*f*_now_).

*Step 1-3: Selecting*. According to the fitness, a standard competition selection strategy is used to get the next population, and then the elite individual is placed into this population.

*Step 1-4: Crossing*. We use the two points' crossing which will be described later to get the next population.

*Step 1-5: Mutation*. We use the standard mutation operator of GA to get the next population (*P*_mut_ is the mutation probability).

*Step 1-6*. Considering that if the maximal number of iterations (*I*_max_) is reached or not, if *I*_max_ is not reached, S1 goes to Step 1-2 or S1 is terminated otherwise. Then, the best solution in the population is our final solution.

The TIN of S1 (P_S1_) is given approximately as the following equation:

(9) $$\begin{array}{c} P_{\text{S}1}=\left(P_{\text{ga}}+P_{\text{ga}}\times P_{\text{mut}}\right)\times I_{\max}. \end{array}$$

### 3.2. Discrete IWO (S2)

IWO, as proposed by Mehrabian and Lucasc [29], is inspired from colonizing weed. In IWO, a feasible solution of a question is mimicked by colonizing weed in paddy fields, which mimics the solution space. In the process of evolution, better weeds produce more seeds and vice versa. The produced seeds are distributed around the weeds, and the step lengths between seeds and weeds are subject to normal distribution. The step lengths are higher in the early stages of IWO and vice versa. Larger step lengths represent global searching in the early stages of IWO and smaller step lengths represent local searching in the later stages conversely. The produced seeds, which will grow into weeds, and the parent weeds are both included in a population. If the population size equals a given size, then preserve it by eliminating worse weeds, or else keep the population size growing until it equals the given size. IWO was first proposed to solve numerical optimization problems, and the normal distribution of produced seeds distributed around the parent weeds is proper for numerical optimization problems. For the purpose of using IWO to solve FJSP, we use a discrete structure to improve IWO and obtain S2. Using integer encoding, feasible solutions (individuals) are discrete points in the solution space. If we force the produced seeds to obey a normal distribution, most new weeds grown from the produced seeds will not be feasible solutions any more. Thus, we propose a strategy called the self-adaptive mutation rule (SMR) which will be described later. Using SMR, the weeds will not produce unfeasible solutions. Moreover, S2 keeps the main characteristics, “global searching in the early stage and local searching in the late stage” of standard IWO, and also adapts to the combinatorial characteristic of FJSP. The steps of S2 are described as follows:

*Step 2-1: Initialization*. A population is initialized as Step 1-1. The initialized population has the minimal population size (*P*_min_).

*Step 2-2: Decoding*. This step is the same as Step 1-2.

*Step 2-3: Computing seed number*. According to the fitness, the seed number (*N*_ind_), which is the number of seeds every weed can produce, is calculated by the following equation:

(10) $$\begin{array}{c} N_{\text{ind}}=\left[\left(f_{\max}-f_{\text{now}}\right)\times \frac{\left(S_{\max}-S_{\min}\right)}{\left(f_{\max}-f_{\min}+1\right)}+S_{\min}\right]. \end{array}$$

In Equation (10) (which ensures that the weed which has lower fitness produces more seeds), *f*_max_ and *f*_min_ denote the maximal fitness and minimal fitness, respectively. *S*_max_ and *S*_min_ denote the maximal seed number and minimal seed number, respectively. The symbol “” denotes rounding.

*Step 2-4:* Spatial *expansion*. Using SMR denoted by Equation (11), the number of integers which need to be mutated in an individual is obtained. Then, the spatial expansion which will be described later is implemented.

(11) $$\begin{array}{c} D_{\text{mut}}=\left[\frac{{\left(I_{\max}-I_{\text{now}}\right)}^{3}\times D_{\max}}{I_{\max}^{3}+D_{\min}}\right]. \end{array}$$

In Equation (11), *D*_mut_ denotes the number of integers which need to be mutated in an individual. *I*_max_ and *I*_now_ denote the maximal number of iterations and the number of iterations in question, respectively. *D*_max_ and *D*_min_ denote the maximal and minimal number of integers which need to be mutated, respectively. Equation (11) ensures that the smaller *I*_now_ is, the larger *D*_mut_ is and vice versa. Thus, in the early stages of S2, *D*_mut_ is large and the “distance” between a seed and parent weed is large, which means that global searching is implemented, and conversely, local searching is implemented in the later stages where *D*_mut_ becomes smaller. Therefore, S2 maintains the main characteristics of IWO through SMR.

*Step 2-5*. Considering whether the maximal population size (*P*_max_) is reached or not, if *P*_max_ is reached, S2 goes to Step 2-2 or goes to Step 2-6 otherwise.

*Step 2-6: Selecting*. According to the fitness, a total number of *P*_max_ weeds which have smaller fitness are selected, obtaining the next population.

*Step 2-7*. Considering whether *I*_max_ is reached or not, if *I*_max_ is not reached, S2 goes to Step 2-2 or S2 is terminated otherwise.

The TIN of S2 (P_S2_) is given by the following equation:

(12) $$\begin{array}{c} P_{\text{S}2}=\frac{P_{\max}\times \left(S_{\max}+S_{\min}\right)}{2\times \left(I_{\max}-C_{1}\right)+C}. \end{array}$$

In Equation (12), *C* is a constant which denotes the number of individuals used until *P*_max_ is reached for the first time. *C*_1_ is the number of iteration times when *P*_max_ is reached for the first time.

### 3.3. Multipopulation GA/IWO (S3/S4)

S3 is obtained using a multipopulation structure to improve S1. We use three populations for S3. The steps of S3 are almost the same as S1 except that S3 has three populations which evolve simultaneously. The three populations are communicating with each other by placing the elite individual of a population into the other two. S4 is obtained similarly to S3. The TIN of S3 and S4 (P_S3_ and P_S4_) is given by Equations (13) and (14), respectively.

(13) $$\begin{array}{c} P_{\text{S}3}=3\times \left(P_{\text{ga}}+P_{\text{ga}}\times P_{\text{mut}}\right)\times I_{\max}, \end{array}$$

(14) $$\begin{array}{c} P_{\text{S}4}=\frac{3\times P_{\max}\times \left(S_{\max}+S_{\min}\right)}{2\times \left(I_{\max}-C_{1}\right)+3\times C}. \end{array}$$

### 3.4. Mixed GA-IWO (S5)

S5 is obtained using the crossover operator of GA to improve IWO. The steps of S5 are described as follows:

*Step 5-1: Initialization*. This step is the same as Step 2-1.

*Step 5-2: Decoding*. This step is the same as Step 2-2.

*Step 5-3: Computing seed number*. This step is the same as Step 2-3.

*Step 5-4: Spatial expansion*. This step is the same as Step 2-4.

*Step 5-5*. Considering whether *P*_max_ is reached or not, if *P*_max_ is reached, S5 goes to Step 5-2 or goes to Step 5-6 otherwise.

*Step 5-6: Selecting*. This step is the same as Step 2-6.

*Step 5-7: Crossing*. This step is the same as Step 1-4.

*Step 5-8*. Considering whether *I*_max_ is reached or not. This step is the same as Step 2-7.

The TIN of S5 (P_S5_) is given by the following equation:

(15) $$\begin{array}{c} P_{\text{S}5}=\frac{P_{\max}\times \left(S_{\max}+S_{\min}\right)}{2\times \left(I_{\max}-C_{1}\right)+C+\left(I_{\max}-C_{1}\right)\times P_{\text{ga}}}. \end{array}$$

### 3.5. Parallel GA-IWO (S6)

S6 is obtained using a parallel structure to improve IWO and GA. S6 has two populations, one of which is processed by S1, and the other is processed by S2. The two populations evolve simultaneously and communicate with each other as in S3.

The TIN of S6 (P_S6_) is given by the following equation:

(16) $$\begin{array}{c} P_{\text{S}6}=\frac{P_{\max}\times \left(S_{\max}+S_{\min}\right)}{2\times \left(I_{\max}-C_{1}\right)+C+I_{\max}\;\times \;\left(P_{\text{ga}}+P_{\text{ga}}\times P_{\text{mut}}\right)}. \end{array}$$

### 3.6. Multistage GA-IWO (S7)

S7 is obtained using a multistage structure to improve IWO and GA. The steps of S7 are described as follows:

*Step 7-1: Initialization*. Like Step 2-1, a population is initialized randomly.

*Step 7-2: Decoding*. This step is the same as Step 2-2.

*Step 7-3: Computing seed number*. This step is the same as Step 2-3.

*Step 7-4: Spatial expansion*. This step is as Step 2-4.

*Step 7-5*. Considering whether *P*_max_ is reached or not, if *P*_max_ is reached, S7 goes to Step 7-2 or goes to Step 7-6 otherwise.

*Step 7-6: Selecting*. According to the fitness of every weed, a total number of *P*_max_ weeds which have smaller fitness are selected and a new population is obtained.

*Step 7-7*. Considering whether the maximal number of iteration times of IWO of one round (*I*_iwo_, which equals 3 in this paper) is reached or not, if *I*_iwo_ is reached, S7 goes to Step 7-8 or S7 goes to Step 7-2 otherwise.

*Step 7-8: Initialization of GA*. To obtain a population for GA, we select the *P*_ga_ better individuals from the population of IWO (*P*_ga_ ≤ *P*_max_) when IWO steps into GA for the first time. On the other hand, we select approximately *P*_ga_/3 better individuals from the population of IWO, and the remaining individuals of GA remain unchanged.

*Step 7-9*. Considering whether *I*_max_ is reached or not, if *I*_max_ is not reached, S7 goes to Step 7-10 or S7 is terminated otherwise. For S7, *I*_max_ is the number of iteration times of GA.

*Step 7-10 Decoding*. This step is the same as Step 1-2.

*Step 7-11 Selecting*. This step is the same as Step 1-3.

*Step 7-12 Crossing*. This step is the same as Step 1-4.

*Step 7-13 Mutation*. This step is the same as Step 1-5.

*Step 7-14*. Considering whether the maximal number of iteration steps of GA of one round (*I*_ga_) is reached or not, if *I*_ga_ is not reached, S7 goes to Step 7-10 or S7 goes to Step 7-2 otherwise.

The TIN of S7 (P_S7_) is given by the following equation:

(17) $$\begin{array}{c} P_{\text{S}7}=\frac{P_{\max}\times \left(S_{\max}+S_{\min}\right)}{2\times I_{\text{iwo}}\times N_{\text{iwo}}+I_{\max}\;\times \;\left(P_{\text{ga}}+P_{\text{ga}}\times P_{\text{mut}}\right)}. \end{array}$$

In Equation (17), *N*_iwo_ denotes how many times S7 goes into the IWO.

## 4. The Seven Algorithms for FJSP

Using the seven algorithms to solve FJSP, the main operators are encoding, decoding, crossing, mutation, and spatial expansion. We describe them in the context of FJSP as follows.

### 4.1. Encoding

We use the integer encoding proposed by Zhang et al. [30] to obtain an individual. The encoding process is divided into two stages, machine encoding and operation encoding. In the process of machine encoding, which is described as a string of integers, the number of integers equals the number of all jobs' operations. The positions and the values of these integers denote the operations and the number of machines that the operations can be processed on, respectively. For example, a machine encoding of the FJSP mentioned in Table 1 is [4 2 5 6 3 1]. There are six integers, and the number of all operations is also six. The position of the third integer represents *O*_22_. Meanwhile, the value of the third integer (5) represents the fifth machine of the candidate machines on which *O*_22_ can be processed, so the integer 5 denotes *M*_6_ rather than *M*_5_. In the process of operation encoding, which is also described as a string of integers, the number of integers is also the same as the number of all jobs' operations. The value of an integer denotes the job number. If the job number is 2 and this job has two operations, then the integer 2 will emerge two times, and so on. For example, an operation encoding the FJSP mentioned in Table 1 is [3 2 1 2 3 3]. The integer 3 emerges three times, which means that job 3 has three operations, and so on. The positions of integers denote the processing sequence. For example, the fourth integer 2 in the encoding above means that *O*_22_ is processed here and so on. The string of integers [4 2 5 6 3 1 3 2 1 2 3 3] represents an individual.

### 4.2. Decoding

A novel decoding method is proposed as follows:

*Step 1*. According to integer encoding, a matrix (**M**′) is obtained. For example, considering the individual [4 2 5 6 3 1 3 2 1 2 3 3], we get **M**′ = [3 1 6 6; 2 1 2 2; 1 1 6 2; 2 2 6 3; 3 2 3 8; 3 3 3 2]. Considering every row of **M**′, the first integer denotes the job number, the second integer the operation number of that job, the third integer the machine number, and the fourth integer the processing time on that machine. For example, the first row [3 1 6 6] denotes that *O*_31_ is processed on *M*_6_, where *P*_316_ is 6.

*Step 2*. According to **M**′, the start time and finish time of each operation are calculated as follows:

- Define a matrix (**M**) and initialize it. **M** is obtained by adding two columns of zeros to **M**′. The integers of the fifth and sixth columns denote *B*_*ijk*_ and *F*_*ijk*_, respectively.
- Considering the first row of **M**′, this operation is the first operation of the corresponding job, and it is the only operation processed on that machine. Thus, this operation can be processed on that machine at the beginning time 0. Consequently, the start time is 0 and the finish time is 0 plus the processing time. For example, *B*_316_ = 0 and *F*_316_ = *B*_316_ + *P*_316_ = 0 + 6 = 6.
- For *l*th row of **M**′, several situations are considered as follows:
   
*Situation I*. If *O*_*ij*_ is the first operation of *J*_*i*_, and *M*_*k*_ is not assigned any operation yet, then *B*_*ijk*_ = 0 and *F*_*ijk*_ = *B*_*ijk*_ +*P*_*ijk*_.
   
*Situation II*. If *O*_*ij*_ is the first operation of *J*_*i*_, and *M*_*k*_ is assigned some operations, then find all idle-time intervals of *M*_*k*_ denoted by [*s*_q_, *e*_q_] (*q* = 1, 2,…). Considering all idle-time intervals one by one, find the first idle-time interval whose interval length is larger than *P*_*ijk*_. Then *B*_*ijk*_ = *s*_q_ and *F*_*ijk*_ = *B*_*ijk*_ +*P*_*ijk*_.
   
*Situation III*. If *O*_*ij*_ is not the first operation of *J*_*i*_, and *M*_*k*_ is not assigned any operation yet, then *B*_*ijk*_ = *F*_*ij*−1*k*_ and *F*_*ijk*_ = *B*_*ijk*_ + *P*_*ijk*_.
   
*Situation IV*. If *O*_*ij*_ is not the first operation of *J*_*i*_, and *M*_*k*_ is assigned some operations, find all idle-time intervals of *M*_*k*_. Then considering all idle-time intervals one by one and considering the relationship between *e*_q_−*s*_q_ and *P*_*ijk*_ and the relationship between *s*_q_ and *F*_*ij*−1*k*_, if *e*_q_−*s*_q_ ≥ *P*_*ijk*_ and *F*_*ij*−1*k*_ ≤ *s*_q_, then *B*_*ijk*_ = *s*_q_ and *F*_*ijk*_ = *B*_*ijk*_ + *P*_*ijk*_; if *e*_q_−*s*_q_ ≥ *P*_*ijk*_ and *F*_*ij*−1*k*_ ≥ *s*_q_ and *e*_q_−*F*_*ij*−1*k*_ ≥ *P*_*ijk*_, then *B*_*ijk*_ = *F*_*ij*−1*k*_ and *F*_*ijk*_ = *B*_*ijk*_ + *P*_*ijk*_; or else, *B*_*ijk*_ is the finish time of the last operation assigned on *M*_*k*_.
- Considering whether all of the rows of **M**′ are considered or not, if all of them are not considered yet, go to (c) or go to the end otherwise.

For example, **M** of the individual mentioned above is [3 1 6 6 0 6; 2 1 2 2 0 2; 1 1 6 2 6 8; 2 2 6 3 8 11; 3 2 3 8 6 14; 3 3 3 2 14 16].

### 4.3. Crossing

**Figure 1:**
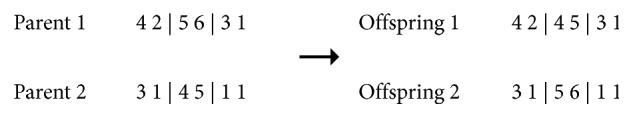
Machine crossing.

Crossing is divided into two stages, machine crossing and operation crossing. In machine crossing, two integers smaller than the number of all operations are generated randomly and two-point crossing is implemented using the two random integers (Figure 1).

**Figure 2:**
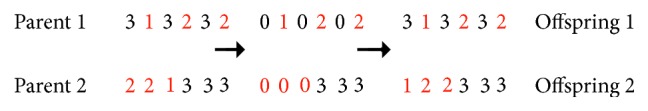
Operation crossing.

In operation crossing, we adopt the POX crossing proposed by Zhang et al. [31]. We choose two individuals randomly, called parent 1 and parent 2, respectively, and the jobs are divided into two groups randomly, called group 1 and group 2, respectively. Then offspring 1 and offspring 2 inherit the integers, which belong to group 1 and group 2, of parent 1 and parent 2, respectively, while preserving the positions of these integers. Offspring 1 and offspring 2 inherit the integers, which do not belong to group 1 and group 2, of parent 2 and parent 1, respectively, preserving the sequence of these integers (Figure 2).

As shown in Figure 2, jobs 1, 2, and 3 are divided into two groups. Group 1 includes jobs 1 and 2 denoted by red integers, and group 2 includes job 3 denoted by black integers.

### 4.4. Mutation

Mutation is divided into two stages, machine mutation and operation mutation. In the process of machine mutation, some individuals are selected according to the mutation probability and some positions for these individuals are chosen randomly. The values of the integers are smaller than the number of the candidate machines, and then these integers are placed in the positions that were chosen in advance. In the process of operation mutation, some individuals are selected randomly according to the mutation probability and the values of two integers are smaller than the number of all operations that are generated randomly. The two generated integers denote two positions and are exchanged with the integers in the selected positions.

### 4.5. Spatial Expansion

According to *D*_mut_ calculated by Equation (10), a new after-expansion individual is obtained through *D*_mut_ times mutations described in *Mutation* and this process is repeated *N*_ind_ times.

## 5. Numerical Simulations

For the purpose of addressing how structures affect different algorithms and how different algorithms respond to the same structure, we use the seven algorithms to solve the five FJSP instances proposed by Kaceam [23].

### 5.1. Simulation Setup

We use S1–S7 to solve the five FJSP instances (denoted by K1–K5).  Table 2 lists the parameters of S1–S7. The symbol “/” in Table 2 denotes parameters that do not exist.

We consider different TINs for different FJSP instances. These TINs are selected based on the TINs not too being large (waste time), and at least one of the seven algorithms can find the optimal value through the largest TIN.  Table 3 lists the different TINs of K1–K5.

### 5.2. Evaluation Indexes Based on TIN

To evaluate S1–S7 fairly, we introduce four evaluation indexes based on TIN as follows: optimal value based on TIN (OVTIN), average value based on TIN (AVTIN), population diversity based on TIN (PDTIN), and premature convergence rate based on TIN (PCRTIN). Give a constant TIN and run the algorithm 20 times independently to obtain 20 solutions of the corresponding FJSP instance, so OVTIN represents the best one of these solutions, and AVTIN is the average of these solutions.

According to the characteristics of integer encoding, the Hamming distance between two individuals is introduced to estimate the population diversity. However, using the average Hamming distance of all pairs in the population is time consuming, so we take a sample including *x* (*x* is 20 in this paper) individuals from the population randomly and the average Hamming distance of this sample is used to represent the population diversity approximately. For the purpose of eliminating the influence of the total number of positions of an individual, the average Hamming distance of the sample is divided by the total number of operations, and the improved average Hamming distance (*H*) is obtained as follows:

(18) $$\begin{array}{c} H=\frac{\sum_{i=1}^{x}\sum_{j=1}^{y}\left(1-\delta \left(a_{ij}^{1},a_{ij}^{2}\right)\right)}{\left(x\times y\right)}. \end{array}$$

In Equation (18), *δ*(.,.) is the Kronecker function; if the two independent variables are equal, then the value of *δ*(.,.) is 1, otherwise 0. The variable *y* denotes the total number of positions in an individual. The variables *a*_*ij*_^1^ and *a*_*ij*_^2^ denote the values of the *j*th position of the *i*th pairs of individuals in the sample. Considering that we run the algorithm 20 times and obtain 20 values of *H*, the PDTIN is their average.

Considering the optimal value of an algorithm for the first time at the *I*_em_th iteration step, the premature convergence rate (*P*_v_) is defined as follows:

(19) $$\begin{array}{c} P_{\text{v}}=\frac{I_{\text{em}}}{I_{\max}}. \end{array}$$

As mentioned above, we run the algorithm 20 times and obtain 20 values of *P*_v_, so PCRTIN is their average.

### 5.3. How Structures Affect Different Algorithms

**Figure 3:**
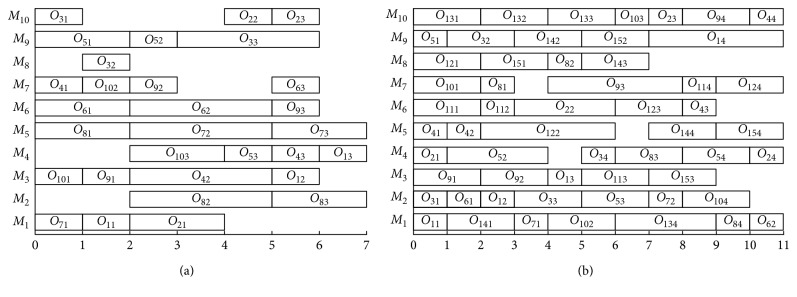
(a) The Gantt chart of K4; (b) for K5.

In this subsection, we discuss how structures affect different algorithms.  Figure 3 gives the Gantt charts of K4 and K5.

**Figure 4:**
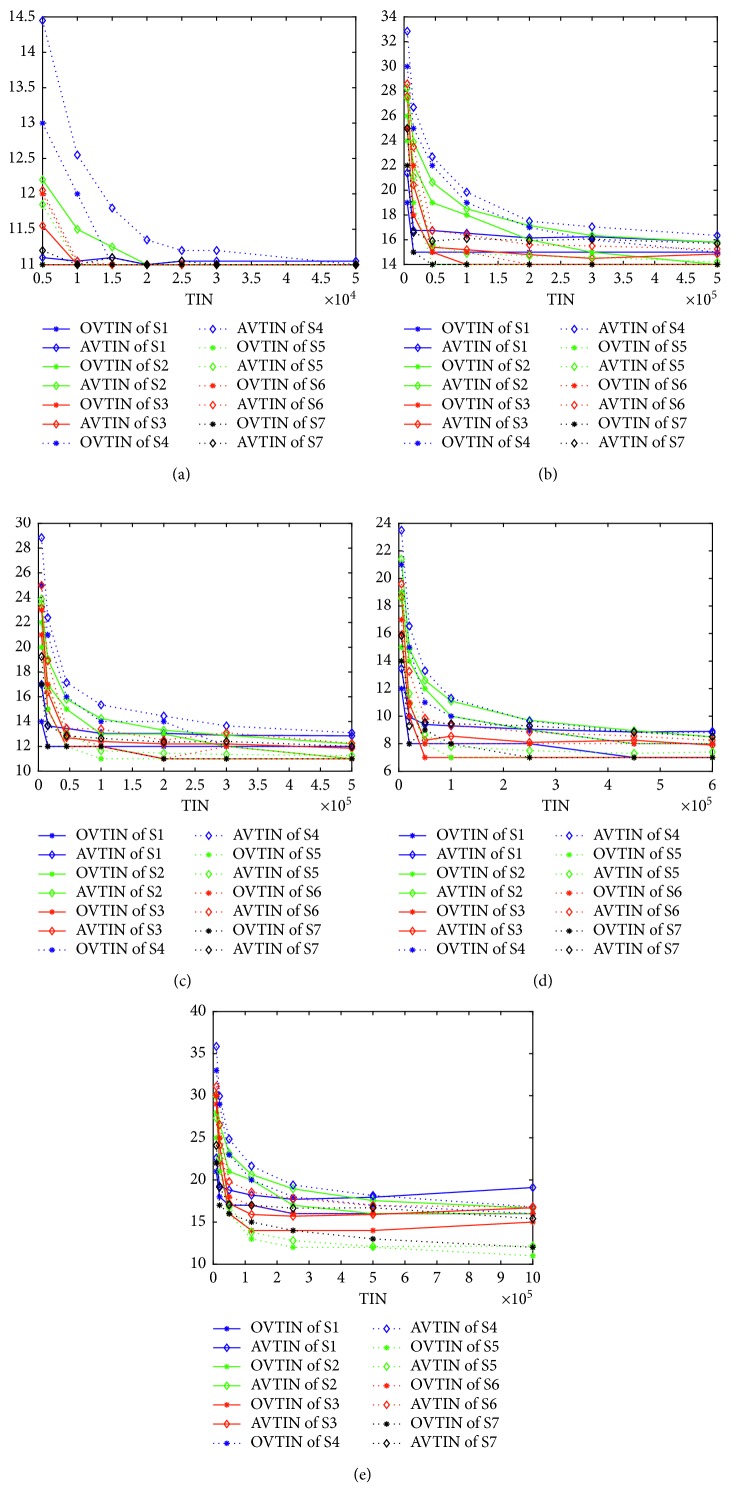
(a) Curves of OVTIN and AVTIN over TIN for K1, (b) for K2, (c) for K3, (d) for K4, and (e) for K5.

Figure 4 gives the curves of OVTIN and AVTIN over TIN for all FJSP instances. The optimal values of K1 to K5 at this point are: 11, 14, 11, 7, and 11.  Figure 4(a), for K1, shows that the performances of all seven algorithms are almost the same. This is mainly because K1 is so simple that all of the seven algorithms can find 11 easily. However, the average performance of S4 is slightly worse than the others. As the problem becomes more complex, the gaps between different algorithms become obviously larger. From Figure 4(b), S1 and S4 ultimately cannot find 14. S5 and S7 can find 14 when the TIN is almost 45,000. However, the average performance of S5 is slightly better than S7. S3 finds 14 when the TIN is almost 100,000 and S6 finds 14 when the TIN becomes 200,000 approximately. Ultimately, S2 finds 14 at 500,000 approximately. From Figure 4(c), S1 and S4 cannot find 11 ultimately. S5 finds 11 at 100,000 approximately and this is the best performance of the seven algorithms. S3, S6, and S7 find 11 at 200,000 approximately which is slightly worse than S5. S2 finds 11 at 500,000 approximately. From Figure 4(d), S2, S4, and S6 cannot find 7. The best of the seven algorithms is S3, which finds 7 at 50,000, rather than S5 that finds 7 at 100,000. S7 finds 7 at 250,000 and S1 follows behind S7.  Figure 4(e) shows that all algorithms cannot find 11 except for S5. The best value found by S7 is 12 when the TIN is almost 1,000,000. The best value found by S3 is 14 and the other four algorithms find 16. In a word, S5 is the best algorithm of the seven algorithms and S7 is second best. Thus, we can conclude safely that the mixed structure is the best structure, at least for IWO and GA, and the multistage structure follows.

**Figure 5 fig5:**
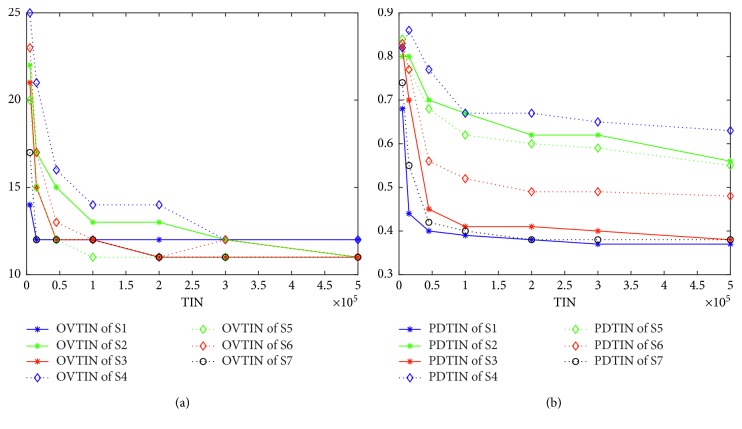
(a) Curves of optimal value over TIN for K3. (b) Curves of population diversity over TIN for K3.

**Figure 6 fig6:**
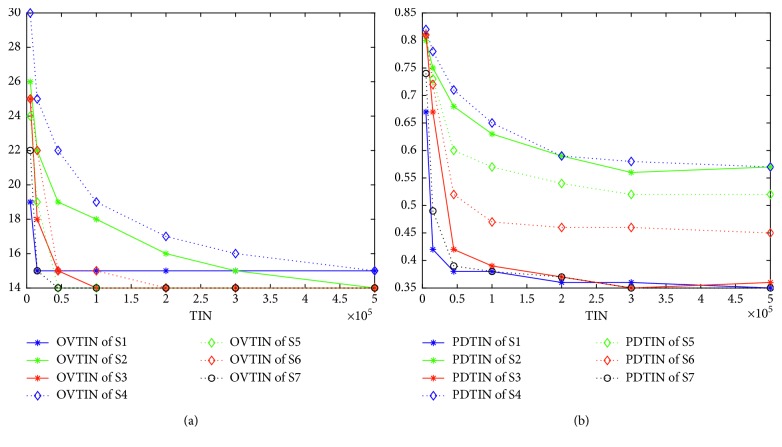
(a) Curves of optimal value over TIN for K2. (b) Curves of population diversity over TIN for K2.

To answer how structures affect different algorithms in detail, we should know how the population diversity affects the performance of an algorithm.  Figure 5 (for K3) gives the relationship between population diversity and performance.  Figure 6 is for K2.  Figure 5(a) gives the curves of OVTIN over TIN, and Figure 5(b) gives the curves of PDTIN over TIN. From these two figures, PDTIN of S1 starts a precipitous decline at the beginning of the curve and drops to 0.4 at 45,000 and changes very slightly from then on. From the curve of OVTIN of S1 shown in Figure 5(a), S1 finds the local optimal value 12 very early (at 15,000) and cannot find 11 ultimately. In contrast, the PDTIN of S2 is always higher than that of S1 and declines slowly. Again from the curve of OVTIN of S2 shown in Figure 5(a), S2 finds 11 at 500,000 although the curve of the OVTIN of S2 declines slowly. From the curve of PDTIN of S6 and that of OVTIN of S6, the value of PDTIN is also larger and declines slowly, so the corresponding algorithm is more likely to find the optimal value. Thus, we can conclude safely that an algorithm is more likely to find the optimal value when the population diversity is larger.  Figure 6 shows the same trend as Figure 5.

**Figure 7 fig7:**
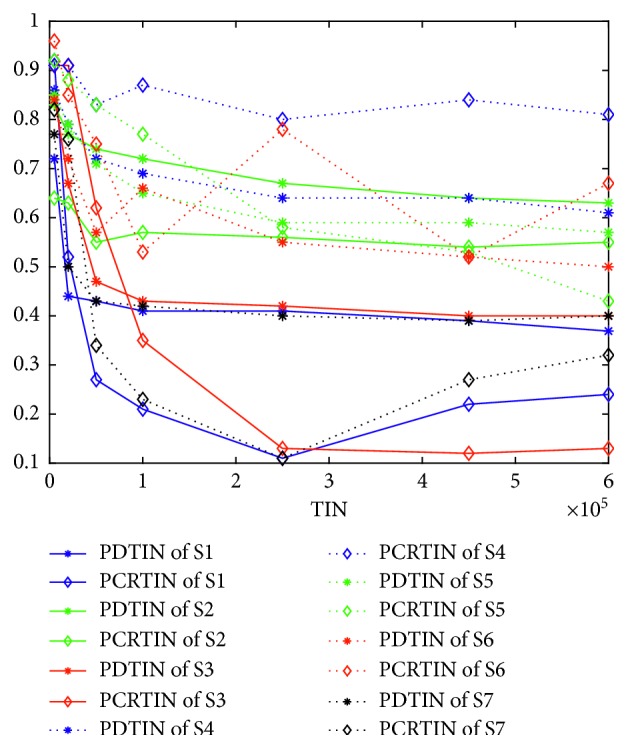
Curves of PCRTIN and PDTIN over TIN for K4.

**Figure 8 fig8:**
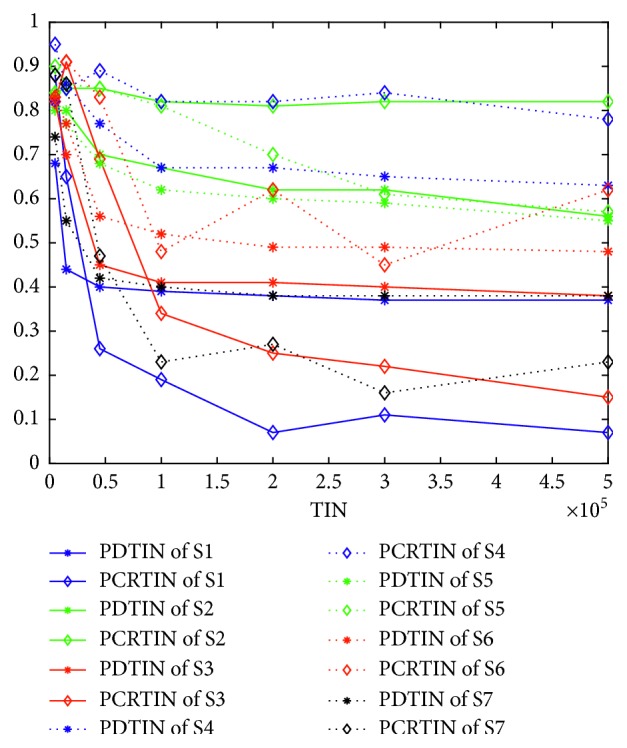
Curves of PCRTIN and PDTIN over TIN for K3.

We propose a hypothesis that when the population diversity of an algorithm is smaller, premature convergence is more likely to occur. To test this hypothesis, we use PCRTIN to evaluate the premature convergence nature of the seven algorithms. Figures 7 and 8 give the curves of PCRTIN and PDTIN over TIN for K4 and K3, respectively. From Figure 7, the PDTIN of S1 declines very fast and remains almost unchanged at 0.39 at 50,000. The PDTIN of S1 shown in Figure 8 also declines very fast and remains almost unchanged at 0.38 at 50,000. The curve of PCRTIN of S1 in Figure 7 shows that at the beginning of the curve, PCRTIN is almost 0.7 which means that 70% of iterations are useful for finding a better solution. As the TIN becomes larger, PCRTIN of S1 drops to 0.2 quickly and remains almost unchanged, meaning that just 20% of iterations are useful and almost 80% of them are useless. As shown in Figure 4(d), S1 finds the local optima 8 at 20,000 and finds 7 at 450,000. In Figures 4(c)–4(e), S1 cannot find its own optimal value but can find local optima very quickly. Thus, we can conclude that S1 is more likely to drop into a local optimum and cannot escape. In Figure 8, almost 85% of iterations are useless for S1. From Figure 7, the values of PDTIN of the seven algorithms in increasing order are S1, S7, S3, S6, S5, S4, and S2. The values of PCRTIN of the seven algorithms in increasing order are S1, S7, S3, S2, S5, S6, and S4. From Figure 8, the values of PDTIN in increasing order are S1, S7, S3, S6, S5, S2, and S4. The values of PCRTIN from in increasing order are also S1, S7, S3, S6, S5, S2, and S4. Thus, we can conclude safely that PDTIN is positively correlated with PCRTIN, meaning that if the population diversity is larger, the corresponding algorithm is more likely to escape from local optimal.

### 5.4. How Different Algorithms Respond to the Same Structure

**Figure 9 fig9:**
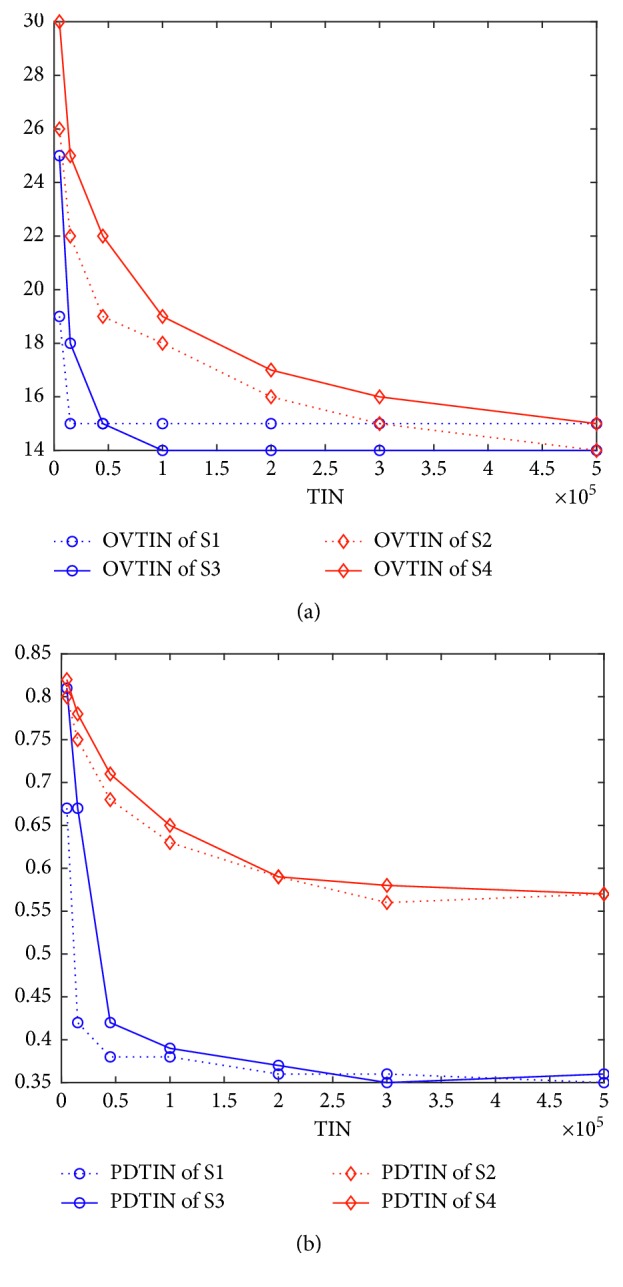
(a) Curves of optimal value over TIN for K2. (b) Curves of population diversity over TIN for K2.

**Figure 10 fig10:**
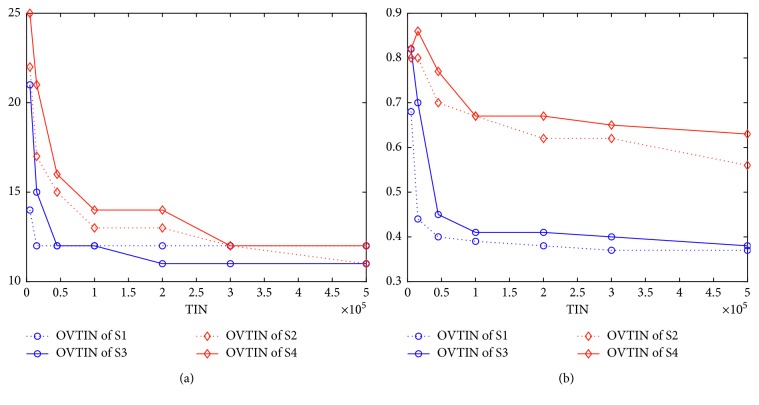
(a) Curves of optimal value over TIN for K3. (b) Curves of population diversity over TIN for K3.

**Figure 11:**
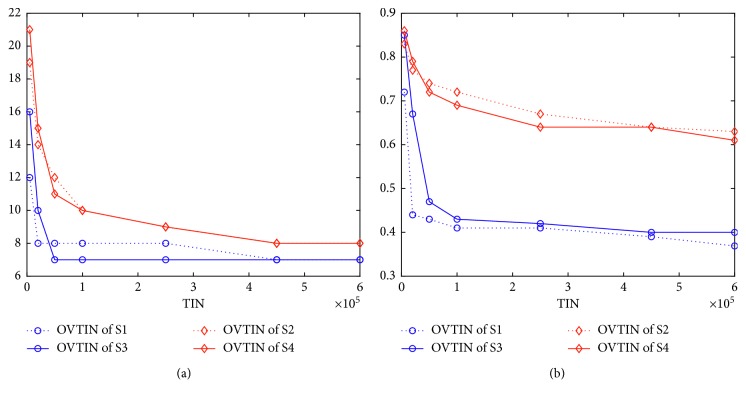
(a) Curves of optimal value over TIN for K4. (b) Curves of population diversity over TIN for K4.

In this subsection, we address how different algorithms respond to the same structure. Using a multipopulation structure, we construct S3 from S1 and S4 from S2. We simply use S1–S4 to address this problem because S5, S6, and S7 are obtained by improving both S1 and S2.  Figure 9 (for K2) illustrates that different algorithms indeed respond to the same structure differently.  Figure 10 is given by solving K3 and Figure 11 for K4. From Figure 9(a), S1 cannot find the optimal value, while S3 (which is improved by the multipopulation structure) can find it quickly at 100,000. From Figure 9(b), the population diversity of S3 becomes larger than S1 through the multipopulation structure. Again from Figure 10(a), we note that S2 can find the optimal value but S4 (which is supposedly improved by multipopulation structure) cannot find the optimal value. From Figure 10(b), the population diversity of S4 becomes larger than S2 through the multipopulation structure.  Figure 10 shows the same trend as Figure 9. Figure 11 shows almost the same trend except that the PDTIN of S4 is slightly smaller than S2. These three figures indicate that the population diversity of an algorithm indeed becomes large through multipopulation, but the performances of different algorithms are different mainly because the population diversity of S1 is very small, so it cannot escape from local optimal. When the population diversity of S3, which is obtained through multipopulation structure, becomes larger, it can escape from local optima and find the global optimal value ultimately. In contrast, the population diversity of S2 itself is very large. Thus, the performance of S4 is not improved by improving population diversity. Therefore, we can conclude that different algorithms indeed have different responses to the same structure. If we want to improve the performance of GA, increasing the population diversity is a good idea, but this is not the case for IWO.

## 6. Conclusions

In this paper, we mainly address two questions: how different structures affect the performance of different intelligent algorithms and how different algorithms respond to the same structure. The simulation results show that different structures significantly affect different algorithms and different algorithms indeed exhibit different performances to the same structure. We obtain several conclusions as follows:

- The performance of the GA can be improved by improving its population diversity and the performance of IWO cannot be improved only by improving the population diversity, so we can use multipopulation structure to obtain better algorithm performance of GA but not for IWO.
- The Hamming distance can represent population diversity properly. When the population diversity is larger, the corresponding algorithm is more likely to escape from local optima. Otherwise, the corresponding algorithm is more likely to exhibit premature convergence.
- The mixed structure is the best structure among the five basic structures studied, at least regarding GA and IWO, followed by the multistage structure. Thus, the mixed structure and the multistage structure should be first considered when selecting improvement strategies to solve FJSP problems.

In the future, other intelligent algorithms will be analyzed using our proposed structures. Additionally, we will evaluate a self-adaptive algorithm based on changing population diversity as the population diversity affects the performance of some algorithms dramatically.

## Figures and Tables

**Table 1:** 3 × 6 FJSP instance.

Job	Operation	Machine					
		*M*_1_	*M*_2_	*M*_3_	*M*_4_	*M*_5_	*M*_6_
*J*_1_	*O*_11_	3	5	7	0	0	2

*J*_2_	*O*_21_	8	2	0	0	0	0
	*O*_22_	5	0	1	2	7	3

*J*_3_	*O*_31_	5	3	2	7	9	6
	*O*_32_	4	6	8	7	2	0
	*O*_33_	0	0	2	0	0	0

**Table 2:** Parameters of S1 to S7.

	*P*_ga_	*P*_mut_	*P*_min_	*P*_max_	*S*_min_	*S*_max_	*D*_min_	*D*_max_
S1	200	0.05	/	/	/	/	/	/
S2	/	/	20	200	1	5	4	20
S3	200	0.05	/	/	/	/	/	/
S4	/	/	20	200	1	5	4	20
S5	/	/	20	200	1	5	4	20
S6	200	0.05	20	200	1	5	4	20
S7	200	0.05	20	200	1	5	4	20

**Table 3:** Different TINs of different FJSP instances.

	*n* × *m*	TIN 1	TIN 2	TIN 3	TIN 4	TIN 5	TIN 6	TIN 7
K1	4 × 5	5000	10000	15000	20000	25000	30000	50000
K2	8 × 8	5000	15000	45000	100000	200000	300000	500000
K3	10 × 7	5000	15000	45000	100000	200000	300000	500000
K4	10 × 10	5000	20000	50000	100000	250000	450000	600000
K5	15 × 10	10000	20000	50000	120000	250000	500000	1000000

## Data Availability

The data is available upon request.

## Supplementary Materials

Supplementary Materials The supplementary materials titled “original data” are the data of Figures 4 [Fig fig5] [Fig fig6] [Fig fig7] [Fig fig8] [Fig fig9] [Fig fig10]–11. For example, “Figure 3.xlsx” in “original data” is the data presented in Figure 4 and so on.
